# An Evolution-Based Screen for Genetic Differentiation between Anopheles Sister Taxa Enriches for Detection of Functional Immune Factors

**DOI:** 10.1371/journal.ppat.1005306

**Published:** 2015-12-03

**Authors:** Christian Mitri, Emmanuel Bischoff, Eizo Takashima, Marni Williams, Karin Eiglmeier, Adrien Pain, Wamdaogo M. Guelbeogo, Awa Gneme, Emma Brito-Fravallo, Inge Holm, Catherine Lavazec, N’Fale Sagnon, Richard H. Baxter, Michelle M. Riehle, Kenneth D. Vernick

**Affiliations:** 1 Institut Pasteur, Unit of Insect Vector Genetics and Genomics, Department of Parasites and Insect Vectors, Paris, France; 2 CNRS, Unit of Hosts, Vectors and Pathogens (URA3012), Paris, France; 3 Department of Chemistry and Molecular Biophysics & Biochemistry, Yale University, New Haven, Connecticut, United States of America; 4 Centre National de Recherche et de Formation sur le Paludisme, Burkina Faso; 5 Department of Microbiology, University of Minnesota, Saint Paul, Minnesota, United States of America; University of California, Davis, UNITED STATES

## Abstract

Nucleotide variation patterns across species are shaped by the processes of natural selection, including exposure to environmental pathogens. We examined patterns of genetic variation in two sister species, *Anopheles gambiae* and *Anopheles coluzzii*, both efficient natural vectors of human malaria in West Africa. We used the differentiation signature displayed by a known coordinate selective sweep of immune genes APL1 and TEP1 in *A*. *coluzzii* to design a population genetic screen trained on the sweep, classified a panel of 26 potential immune genes for concordance with the signature, and functionally tested their immune phenotypes. The screen results were strongly predictive for genes with protective immune phenotypes: genes meeting the screen criteria were significantly more likely to display a functional phenotype against malaria infection than genes not meeting the criteria (p = 0.0005). Thus, an evolution-based screen can efficiently prioritize candidate genes for labor-intensive downstream functional testing, and safely allow the elimination of genes not meeting the screen criteria. The suite of immune genes with characteristics similar to the APL1-TEP1 selective sweep appears to be more widespread in the *A*. *coluzzii* genome than previously recognized. The immune gene differentiation may be a consequence of adaptation of *A*. *coluzzii* to new pathogens encountered in its niche expansion during the separation from *A*. *gambiae*, although the role, if any of natural selection by *Plasmodium* is unknown. Application of the screen allowed identification of new functional immune factors, and assignment of new functions to known factors. We describe biochemical binding interactions between immune proteins that underlie functional activity for malaria infection, which highlights the interplay between pathogen specificity and the structure of immune complexes. We also find that most malaria-protective immune factors display phenotypes for either human or rodent malaria, with broad specificity a rarity.

## Author Summary

*Anopheles gambiae* and *Anopheles coluzzii* are the primary mosquito vectors of human malaria in West Africa. Both of these closely related species efficiently transmit the disease, although they display ecological differences. Previous work showed that *A*. *coluzzii* displays distinct genetic patterns in genes important for mosquito immunity. Here, we use this genetic pattern as a filter to examine a panel of potential immune genes, and show that the genetic pattern is strongly predictive for genes that play a functional role in immunity when tested with malaria parasites.

## Introduction

Malaria remains a serious global public health concern. In Africa, members of the *Anopheles gambiae* species complex are primary mosquito vectors of the human malaria parasite, *Plasmodium falciparum*. The *A*. *gambiae* complex consists of at least eight morphologically identical sibling species. Previous studies have characterized population structure of the *A*. *gambiae* complex, focusing particularly on the sympatric subgroups originally named the M and S molecular forms of *A*. *gambiae* sensu stricto, which were renamed as *A*. *coluzzii* and *A*. *gambiae*, respectively [1]. *A*. *coluzzii* is apparently the derived form, and has adapted to different ecological conditions as compared to the ancestral form, *A*. *gambiae* [2–4]. The two groups are partially reproductively isolated [5–8]. Most genome-wide genetic variation is shared between *A*. *coluzzii* and *A*. *gambiae*. Genomic regions of differentiation were described, termed speciation islands [9–11], although the role of these islands in population differentiation or speciation currently remains unresolved [12, 13].

Two unlinked loci outside of the described speciation islands contain the immune genes *APL1* and *TEP1*, which display significantly reduced genetic diversity in *A*. *coluzzii* as compared to *A*. *gambiae*, resulting from an apparent coordinate selective sweep in *A*. *coluzzii* [14, 15]. The selective pressures underlying the loss of diversity at these immune loci in *A*. *coluzzii* are not known, but are most plausibly based on exposure of *A*. *coluzzii* to distinct pathogen profiles in the newly colonized niches [14–16]. *APL1* is a paralogous gene family encoding three leucine-rich repeat (LRR) factors [17], and *TEP1* encodes a mosquito complement-like factor [18]. *TEP1* and the *APL1* family display protective activity against *Plasmodium* in functional assays [19–21], although an association phenotype has not yet been demonstrated for genetic variants of these genes in the outbred population. The reason for the multilocus coordinate nature of the *APL1-TEP1* selective sweep is unknown. However, TEP1 and the APL1 paralog APL1C, along with another LRR protein, LRIM1, form a ternary immune complex that is required for protection against the rodent malaria parasites, *P*. *berghei* and *P*. *yoelii* [19, 20, 22, 23]. Thus, the subunits could be under evolutionary constraint to maintain biochemical interaction in an essential functional complex.

We reasoned that *APL1* and *TEP1* may not be the only *A*. *coluzzii* genes responding to such putative strong, recent pathogen selection. We designed a screen based on the pattern of *APL1* and *TEP1* population genetic differentiation in order to identify other such functional immune factors. The screen is phenotype-free, based solely upon the strongly divergent patterns of nucleotide diversity observed across sister taxa at these known immune loci. Population and evolutionary genetics is an underutilized line of evidence for ascertainment of candidates for functional studies. An analogous phenotype-free gene filter using signatures of natural selection successfully discovered new functionally confirmed immune elicitors produced by bacterial phytopathogens of *Arabidopsis* [24]. Analysis of positive selection of the viral restriction factor TRIM5α in different primate lineages highlighted a 13-amino acid protein domain that, when functionally tested, explained some of the difference in cellular susceptibility to HIV infection between rhesus and human [25]. In an indirect screen, the forkhead box P2 (FOXP2) transcription factor, a target of strong positive selection in recent human evolution, was used as bait to pull down interacting target proteins, which were queried for and displayed similar patterns of positive selection as the bait [26].

We analyzed population sequence data from *A*. *gambiae* and *A*. *coluzzii* for similarity to the pattern of divergence displayed by the *APL1* and *TEP1* genes. Agreement with the differentiation signal trained on the swept genes was significantly correlated with functional immune activity, as determined functionally by gene silencing and infection challenge with rodent and human *Plasmodium*. Multiple novel malaria-protective mosquito factors were identified. We previously described distinct specificities of the LRR proteins APL1C and APL1A for different pathogen classes [21]. Here, we find that most tested factors display protection specificity against either human or rodent malaria classes, but not both. Binding interactions are newly described between the LRR proteins APL1A, APL1B and LRIM1 using in vitro cellular assays. The results suggest that a combinatorial repertoire of interacting subunits together form a range of functionally diverse immune complexes. Thus, signatures of population differentiation modeled after a coordinate epistatic sweep of immune genes in *Anopheles* in nature can serve as an efficient filter for immune genes that protect against different pathogen classes. The application of these ascertainment criteria to large population-based sequence datasets of these two *Anopheles* species should allow efficient targeting of labor-intensive immune functional assays to the most promising candidates.

## Results

### An index of population differentiation generates three candidate gene clusters

We analyzed population-based sequence datasets for two criteria of genetic differentiation modeled on the *APL1* and *TEP1* genes: i) low ratio of *A*. *coluzzii* to *A*. *gambiae* nucleotide diversity, and ii) high pairwise Fst between *A*. *coluzzii* and *A*. *gambiae*. Sequence data was previously obtained for 26 genes from wild *A*. *coluzzii* and *A*. *gambiae* mosquitoes from Burkina Faso (S1 Table) and analysis of population genetic parameters and selection signatures for 23 of these genes was previously presented [27]. The genes were chosen as candidates based on bioinformatic assignment to gene families with potential immune function. Control genes did not belong to potential immune families. Four genes previously reported to display protective function against *Plasmodium* were included (*APL1A*, *APL1C*, *LRIM1*, and *TEP1*).

Sequences were tested for differentiation between *A*. *coluzzii* and *A*. *gambiae* based on the ratio of nucleotide diversity pi (π) in *A*. *coluzzii* relative to *A*. *gambiae*, and pairwise Fst. On a genome-wide basis, *A*. *coluzzii* and *A*. *gambiae* are equivalent for both parameters. Overall nucleotide diversity in *A*. *coluzzii* is only slightly lower than *A*. *gambiae* [27–30], and Fst between *A*. *coluzzii* and *A*. *gambiae* is low across the genome, with the exception of the centromeric speciation islands, which display high levels of differentiation between the mosquito groups. None of the 26 analyzed genes are located in the speciation islands. This analysis detects genes that show asymmetric patterns of diversity, specifically lower pairwise diversity in *A*. *coluzzii* as compared to *A*. *gambiae*, and does not test for positive selection.

**Fig 1:**
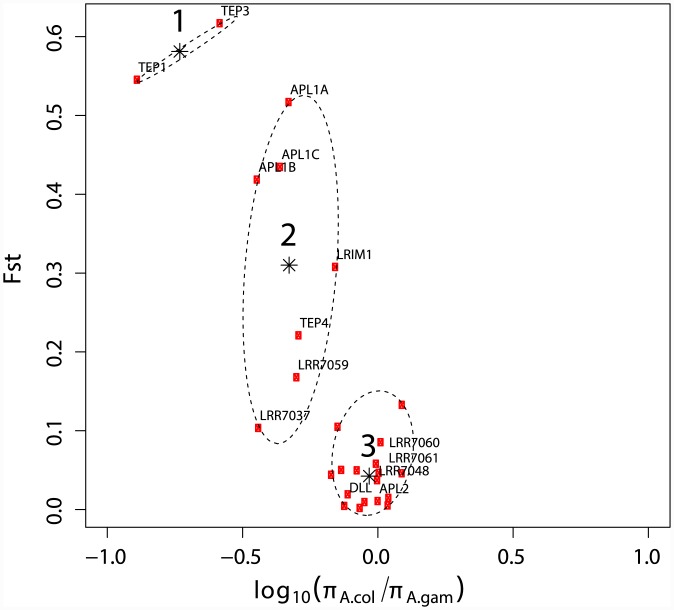
Population genetic parameters partition *Anopheles coluzzii* and *A*. *gambiae* candidate genes into discrete clusters. For 26 genes sequenced from wild *A*. *coluzzii* and *A*. *gambiae* mosquitoes from Burkina Faso, scatterplot indicates the log-ratio of nucleotide diversity, π, for *A*. *coluzzii* / *A*. *gambiae* (x-axis) an Fig 1. Population genetic parameters partition Anopheles coluzzii and A. gambiae candidate genes into discrete clusters. For 26 genes sequenced from wild A. coluzzii and A. gambiae mosquitoes from Burkina Faso, scatterplot indicates the log-ratio of nucleotide diversity, π, for A. coluzzii / A. gambiae (x-axis) and pairwise Fst between A. coluzzii and A. gambiae (y-axis). Negative values for log-ratio of π indicate greater diversity in A. gambiae positive values greater diversity in A. coluzzii, and a value of 0 indicates no difference in nucleotide diversity between A. coluzzii and A. gambiae. The names of genes subsequently tested by functional analyses are shown, for others see S1 Table. Dashed ovals indicate unbiased hierarchical clusters identified by the method of Ward based on minimized within-cluster variance, with optimal number of clusters k = 3 as determined by the Krzanowski-Lai index (S1 Fig), stars indicate cluster centroids.d pairwise Fst between *A*. *coluzzii* and *A*. *gambiae* (y-axis). Negative values for log-ratio of π indicate greater diversity in *A*. *gambiae* positive values greater diversity in *A*. *coluzzii*, and a value of 0 indicates no difference in nucleotide diversity between *A*. *coluzzii* and *A*. *gambiae*. The names of genes subsequently tested by functional analyses are shown, for others see S1 Table. Dashed ovals indicate unbiased hierarchical clusters identified by the method of Ward based on minimized within-cluster variance, with optimal number of clusters k = 3 as determined by the Krzanowski-Lai index (S1 Fig), stars indicate cluster centroids.

Unbiased hierarchical clustering by the method of Ward [31] was used to group the calculated gene differentiation values by minimizing the total within-cluster variance (Fig 1). All possibilities of cluster numbers from 2–15 were examined, and the Krzanowski-Lai index [32, 33] unambiguously indicated that the optimal number of clusters with minimized within-cluster variance was k = 3 (S1 Fig). Clusters 1 and 2 together contain nine genes that display genetic differentiation between *A*. *coluzzii* and *A*. *gambiae*, and capture all four known anti-*Plasmodium* factors included in the current study: *APL1A* [21], *APL1C* [34], LRIM1 [35], and *TEP1* [18]. Cluster 3 contains 17 genes that are not genetically differentiated between *A*. *coluzzii* and *A*. *gambiae*.

As indicated by the π ratio data (S1 Table), in all cases the pairwise diversity for Cluster 1 and 2 genes is higher in *A*. *gambiae*. For both species, the genes in Cluster 3 show levels of diversity that are comparable with their own Clusters 1 and 2. For Clusters 1 and 2, the range of π for *A*. *coluzzii* is (0.001–0.03) and for *A*. *gambiae* is (0.004–0.07) for Cluster 3 these ranges are *A*. *coluzzii* (0.005–0.03) and *A*. *gambiae* (0.006–0.03). Detection of differentiation between species for genes in Clusters 1 and 2 is thus not a consequence of low overall diversity in *A*. *coluzzii*, because diversity levels are not different across clusters in *A*. *coluzzii* (comparison of π values from S1 Table, Clusters 1 and 2 versus Cluster 3, WMW, p = 0.178) or *A*. *gambiae* (WMW, p = 0.850).

These results suggested that parameters of population genetic differentiation of *A*. *coluzzii* from the ancestral form, *A*. *gambiae*, could be informative for immune function, at least against malaria parasites. We functionally tested protective activity against *Plasmodium* for all nine genes from differentiated Clusters 1 and 2, and five randomly chosen genes from non-differentiated Cluster 3, using RNAi-mediated gene silencing followed by *Plasmodium* infection challenge. All 14 genes were phenotyped for effect upon a panel of *Plasmodium* species, including the human malaria *P*. *falciparum*, and at least one rodent malaria parasite. The rodent parasite *P*. *berghei* produces normal oocysts, while *P*. *yoelii* produces mostly melanized oocysts, which scores an additional immune-related phenotype [34, 36–38]. Comprehensive data for all gene-parasite phenotypes and controls are presented in S2 Table, and selected phenotype analyses are shown in the figures to illustrate biological points. Cluster membership of candidates is listed in S1 Table and in the summary figure of candidate gene differentiation and function.

### *Plasmodium* protection phenotypes of leucine-rich repeat (LRR) proteins

**Fig 2:**
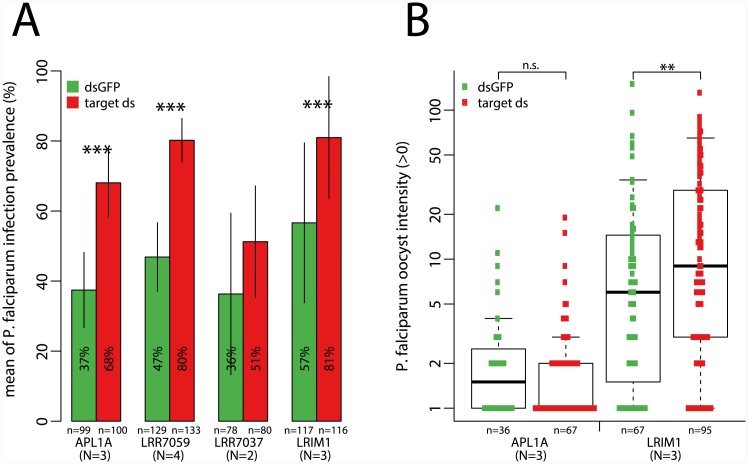
Leucine-rich repeat (LRR) proteins can control a binary switch for establishment of *P*. *falciparum* infection. **A.** LRR proteins *APL1A*, *LRR7059* and *LRIM1* influence permissiveness for initial infection, regardless of subsequent parasite intensity, reflected as increased *P*. *falciparum* oocyst infection prevalence after loss of gene function. **B.** *APL1A*, *LRR7059* and *LRR7037* do not influence oocyst infection intensity (for clarity only *APL1A* is shown, data for all genes available in S2 Table). However, *LRIM1* function also limits the efficiency of parasite development in established infections, reflected as increased intensity of *P*. *falciparum* oocysts after *LRIM1* silencing. Graph labels and statistical tests for this and subsequent figures: tests of infection prevalence indicate mean infection prevalence within histogram bars, error bars indicate standard error. For tests of infection intensity, the y-axis is logarithmic to depict both low and high intensity phenotypes, boxplots delineate the first and third quartile, median is indicated within the box, and error bars are 1.5 times the interquartile range. Sample sizes (N) indicate the number of independent replicate experiments, (n) the total number of mosquitoes dissected across replicates. All statistical differences were first tested independently within replicates (individual p-values, S2 Table), and only if individual replicates showed a consistent direction of change, individual p-values were combined using the meta-analytical approach of Fisher. (Significance levels of Fisher-combined p-values: n.s., not significant; * p-value<0.05; ** p-value <0.01; *** p-value <0.001).

We first evaluated protection mediated by LRR proteins against *P*. *falciparum*. Silencing of either *LRR7059* or *APL1A* by treatment of mosquitoes with specific double-stranded RNA (dsRNA) increased the proportion of *P*. *falciparum*-infected mosquitoes (oocyst infection prevalence) but did not influence oocyst numbers (oocyst infection intensity; Fig 2A and S2 Table), while ablating *LRIM1* function increased infection intensity as well as prevalence (Fig 2A and 2B), and silencing of *LRR7037* affected neither phenotype. Thus, two protective phenotypes are evident, control over infection prevalence, which is a binary permissive switch for establishment of infection regardless of subsequent parasite numbers, and control over infection intensity, which regulates efficiency of development among established infections. The phenotype of *APL1A* is consistent with published work [21]. *LRIM1* is unusual in protecting against both human and rodent malaria species (*P*. *falciparum*, shown here; rodent malaria previously reported in [35]).

**Fig 3:**
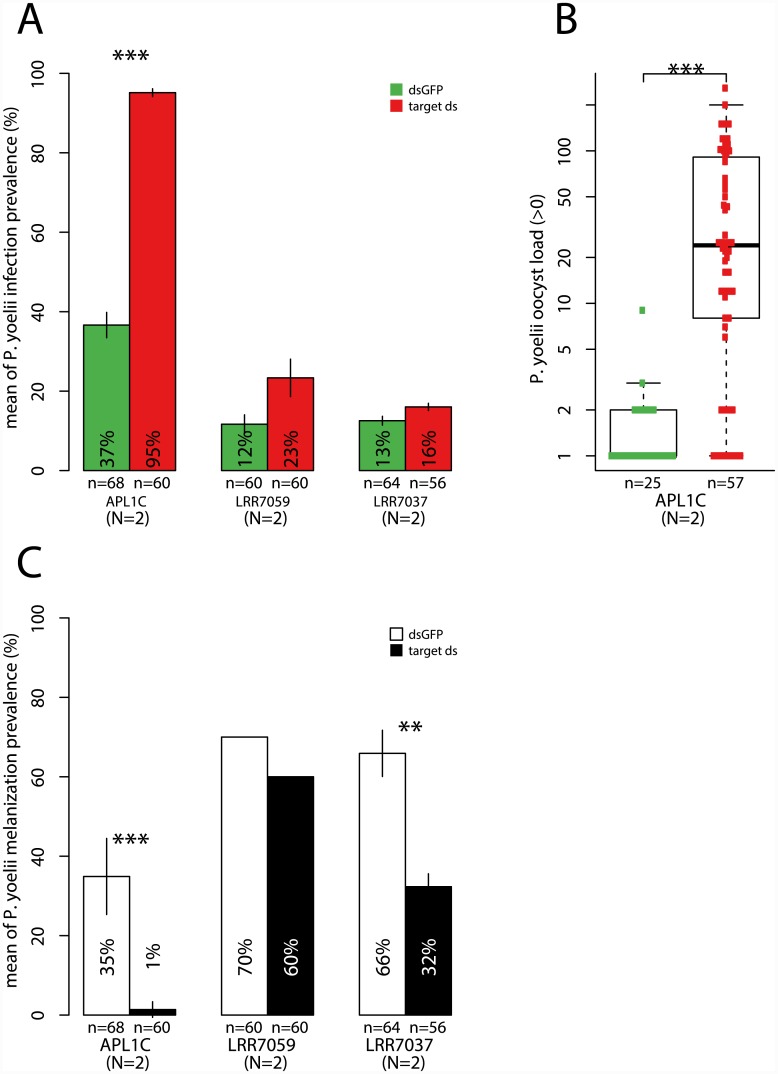
Leucine-rich repeat (LRR) proteins play contrasting roles in the choice between normal and melanized parasite fates. **A**. The activity of APL1C but not LRR7037 restricts permissiveness of mosquitoes to *P*. *yoelii* infection, measured as oocyst infection prevalence. **B.** APL1C function also limits the efficiency of parasite development within infected mosquitoes, measured as infection intensity. LRR7059 and LRR7037 have no effect (S2 Table). **C.** Loss of function of either APL1C or LRR7037 is accompanied by reduced levels of parasite melanization. In the absence of APL1C activity, the non-melanized parasites appear to develop normally (compare C with A), while in the absence of LRR7037 activity, the non-melanized parasites nevertheless are apparently still killed. Graph labels and statistical tests as in Fig 2 legend.

We then measured LRR-mediated protection using the rodent malaria parasite, *P*. *yoelii*, which allows measurement of two phenotypes, normal and melanized oocyst levels, in the same mosquitoes. Silencing of *APL1C* allowed greater prevalence and intensity of normal oocysts (Fig 3A and 3B), as well as caused the near-absence of melanized parasites (Fig 3C). The figure depicts two distinct phenotypes measured in the same infections: panel A shows the effect of the gene upon prevalence of normal oocysts, while panel C shows, for the same mosquitoes, the prevalence of melanized oocysts. Individual mosquitoes can carry both oocyst outcomes. APL1C activity is required for *P*. *yoelii* melanization, whereas loss of APL1C activity permits normal parasite development. Similar to *APL1C*, silencing of *LRR7037* decreased the proportion of mosquitoes carrying melanized parasites (Fig 3C). However, in distinction to *APL1C*, LRR7037 did not influence levels of prevalence or intensity of normal oocysts. Thus, it appears that the parasites rescued from melanization by silencing of *APL1C* developed normally (95% infection prevalence, panel A), while the two phenotypes are decoupled for *LRR7037*, because decreased levels of parasite melanization were not reflected in increased normal development (no change, panel A). The results suggest that LRR7037 function promotes the downstream melanization of already-killed parasites, without contributing to parasite killing, while APL1C activity appears to have both roles, required for killing but also with an indispensable role in melanization.

### *Plasmodium* protection mediated by thioester proteins (TEP)

**Fig 4:**
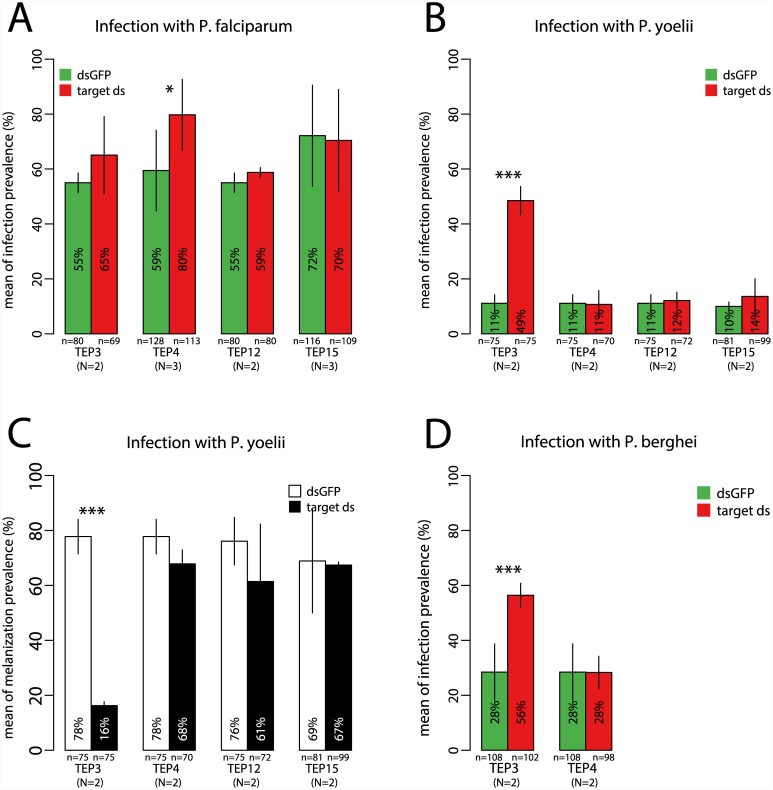
Thioester proteins (TEP) display distinct protection specificities against human and rodent *Plasmodium*. **A.** Of the TEP genes assayed, only *TEP4* displays activity against *P*. *falciparum* infection. **B.** Conversely, only the activity of TEP3 is required for protection against *P*. *yoelii* normal oocysts. **C.** Silencing of *TEP3*, which leads to elevated infection prevalence for normal oocysts, simultaneously diminishes the prevalence of melanized *P*. *yoelii* parasites. **D.** Similar to (B), activity of *TEP3* is required for protection from a different rodent malaria species, *P*. *berghei*. Two *TEP* genes located on chromosome 3R, *TEP12* and *TEP15*, part of a *TEP* clade evolutionarily distinct from *TEP3* and *TEP4* (S2 Fig), did not display protective activity against any of the *Plasmodium* species. Graph labels and statistical tests as in Fig 2 legend.

A family of 15 genes encodes the thioester protein (TEP) family, which are immune complement analogs [39]. The three TEP genes analyzed by population sequencing (*TEP1*, *TEP3* and *TEP4*) displayed genetic differentiation between *A*. *gambiae* and *A*. *coluzzii* (Fig 1). *TEP1* was previously reported to protect *A*. *gambiae* against *P*. *falciparum* and the rodent malaria *P*. *berghei* [18, 40]. Here, we also found that *TEP4*, but not *TEP3*, was protective against *P*. *falciparum*, influencing infection prevalence but not oocyst intensity (Fig 4A, S2 Table). In contrast, *TEP3*, but not *TEP4*, displayed protective function against the rodent parasite *P*. *yoelii*, influencing the infection prevalence for both normal and melanized parasites (Fig 4B and 4C) with a phenotype similar to APL1C as in Fig 3.

The observation that *TEP3* and *TEP4* display reciprocal protective specificity against rodent and human malaria parasites, respectively, mirrors the parasite class discrimination demonstrated by *APL1C* and *APL1A* [21]. We evaluated the phenotypes of TEP3 and TEP4 activity using another rodent malaria parasite, *P*. *berghei*, with results concordant with published work [41]. *TEP3* is similarly protective against *P*. *berghei* (Fig 4D), thus exhibiting the same spectrum of protection as *APL1C*. *TEP4*, protective against *P*. *falciparum* but not *P*. *yoelii*, is also not protective against *P*. *berghei* (Fig 4D), thus displaying the same protection profile demonstrated by *APL1A*. These observations suggest a potential pairwise interaction between members of the LRR and TEP families in mediating protection, where TEP4 could interact with APL1A and TEP3 with APL1C. Consistent with this possibility, APL1C was previously shown to physically interact with both TEP1 [20, 22] and TEP3 [41, 42].

The *TEP* genes are grouped at two genomic locations, and phylogenetic analysis indicates that these represent distinct evolutionary lineages of *TEP* paralogs (S2 Fig). The genes for paralogs with malaria protective function, *TEP1* [18, 40], *TEP3* and TEP4 (shown above), are located on the left arm of chromosome 3 (Chr3L). To compare genes from the TEP lineage on Chr3R, which were not part of the above analysis, we made an *ad hoc* choice of two Chr3R *TEP* genes, *TEP12* and *TEP15*, for functional analysis. The two genes showed no protective phenotype for *P*. *falciparum* (Fig 4A) or *P*. *yoelii* (Fig 4B and 4C). *TEP9*, which is also found in the Chr3L branch with the three known protective *TEPs*, was reported to biochemically interact with the APL1C/LRIM1 heterodimer, hinting at an as yet untested role for it in protection to *Plasmodium* [41]. Taken together, these results suggest that the two divergent *TEP* family branches have evolved distinct functions with regard to immunity.

### Signature of population differentiation is highly informative for malaria-protective phenotype

**Fig 5:**
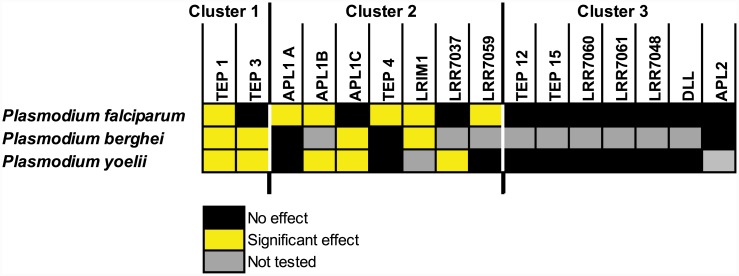
Summary of candidate gene differentiation and immune function. Genes differentiated between *A*. *gambiae* and *A*. *coluzzii* populations (Clusters 1 and 2) and non-differentiated genes (Cluster 3; see Fig 1) were silenced, followed by challenge with human (*P*. *falciparum*) or rodent (*P*. *berghei* and *P*. *yoelii*) malaria parasites. Gene phenotypes for protection against *Plasmodium* infection outcome are indicated by box color. Genes are arranged by Cluster membership and are classified as having a significant effect on parasite infection (yellow) or no significant effect (black). Gene-parasite combinations not tested are indicated in gray. Genes in differentiated Clusters are significantly enriched for *Plasmodium*-protective phenotype as compared to non-differentiated genes (p = 0.0005, see Results). Detailed data for all tests in S2 Table.

Finally, we also functionally assayed 5 candidates belonging to the non-differentiated Cluster 3. Four LRR-coding genes (*LRR7060*, *LRR7061*, *LRR7048*, *APL2*), a family with potential immune function, and one developmental gene without predicted immune function (*Distal-less*) were silenced and mosquitoes were challenged separately with *P*. *falciparum* and *P*. *yoelii*. None of these non-differentiated genes displayed immune function against any of the *Plasmodium* species tested (Fig 5). In contrast, all of the genes in Clusters 1 and 2, which display population-level differentiation between subgroups, were protective against one or more *Plasmodium* species. Thus, screening for a signature of genetic differentiation derived from the coordinate selective sweep in *A*. *coluzzii* efficiently enriched for genes that display a protective function against *Plasmodium* (Fisher’s Exact Test comparing the ratio of [9 protective to 0 non-protective genes in differentiated Clusters 1 and 2], to [0 protective to 5 non-protective genes in non-differentiated Cluster 3] is highly significant, p = 0.0005). Additional gene silencing assays beyond the n>4400 mosquitoes tested in the current study would be necessary to determine profiles of protection against other pathogens. However, the results here indicate that genetic divergence between sympatric *A*. *gambiae* and *A*. *coluzzii*, presumably a consequence of differential pathogen selection pressure, is correlated with consistent and predictable differences in immune function, as detected using pathogen challenge by two classes of malaria parasites. In these two diverging mosquito taxa, the clustering criteria applied to population sequences would appear to be a useful filter to prioritize candidate genes for downstream functional examination.

### Biochemical interaction underlies protective function

LRIM1 function protects mosquitoes against *P*. *falciparum* (Fig 2), in addition to rodent malaria [35]. LRIM1 is a subunit of an immune complex that includes APL1C [19]. APL1C mediates specific protective activity against rodent but not human malaria [21]. Based on these observations, we hypothesized that LRIM1 may additionally interact in a non-APL1C immune complex, perhaps with the *P*. *falciparum*-protective APL1 paralog, APL1A. Because LRIM1 interacts with the coiled-coil domain of APL1C, we queried LRIM1 for biochemical interaction with the other coiled-coil domain APL1 paralogs, APL1A and APL1B.

**Fig 6:**
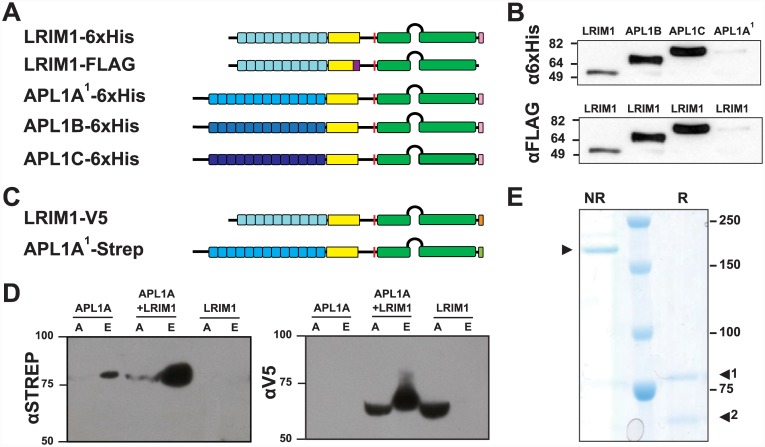
LRIM1 can form protein complexes with all members of the APL1 family. **A.** Structure of expression plasmids used for co-immunoprecipitation of proteins expressed in *Trichoplusia* cells. Fusion tags: purple, FLAG tag; pink, 6xHis tag. Small boxes represent LRR repeats; yellow, Cys-rich region; green, coiled-coil (CC) domain with helix-loop-helix region between CC domains. Additional structural details in [23]. **B.** FLAG-tagged LRIM1 was used to pull down 6xHis-tagged LRIM1, APL1A1, APL1B and APL1C. Western blots were performed with anti-6xHis/HRP (top panel) and anti-FLAG/HRP (bottom panel) to detect the 6xHis-tagged co-precipitated proteins and FLAG-tagged LRIM1, respectively. **C.** Expression plasmids used for pull-downs of proteins expressed in *Drosophila* S2 cells. Fusion tags: orange, V5 tag; light green, Strep tag; other protein features as in (A). **D.** S2 cells were transfected with Strep-tagged APL1A, V5-tagged LRIM1, or co-transfected with both plasmids. Cell extracts were purified by Strep-tag column affinity, and analyzed by Western blot using anti-Strep or anti-V5 antibodies. Lane labels: A, cell extract applied to Strep column; E, eluted fraction from Strep column. **E.** Coomassie-stained SDS-PAGE of extract from co-transfected cells (APL1A-Strep+LRIM1-V5) after elution from Strep column. Lane NR, sample non-reduced, arrow indicates APL1A/LRIM1 heterodimer; lane R, sample reduced, arrow 1 indicates APL1A-Strep monomer, arrow 2 LRIM1-V5 monomer.

6xHis-tagged LRIM1, APL1B, APL1C and APL1A were co-expressed with FLAG-tagged LRIM1 in lepidopteran *Trichoplusia ni* cells and co-immunoprecipitation assays were performed from the conditioned media (Fig 6A). Both APL1B and APL1C were efficiently co-precipitated with LRIM1-FLAG (Fig 6B), revealing that LRIM1 also interacts with APL1B. APL1A is expressed inefficiently in this insect cell system, but suggested a possible interaction with LRIM1 (Fig 6B and [23]). Therefore, we used *Drosophila* S2 cells to co-express recombinant APL1A bearing a C-terminal Strep-tag, and LRIM1 with a C-terminal V5-tag (Fig 6C). The proteins were secreted into the culture medium through their endogenous signal peptide and purified by Strep-tag column affinity. The eluted fractions of the column were analyzed on Western blots using anti-Strep and anti-V5 antibodies. APL1A is efficiently expressed in the *Drosophila* cells (Fig 6D). When LRIM1 and APL1A were co-expressed, both proteins were co-purified in the fraction retained by the Strep-tag column, indicating that LRIM1 interacts with and forms a complex with APL1A (Fig 6D). As expected, when transfected alone, the V5-tagged LRIM1 protein was detected only in the flow-through fraction of the Strep-tag column and Strep-tagged APL1A was retained. The purified APL1A/LRIM1 complex migrated with a 180 kDa molecular mass in non-reducing SDS-PAGE, whereas in reduced conditions the eluted complex was separated as two proteins corresponding to predictions for APL1A (76 kDa) and LRIM1 (60 kDa) (Fig 6D). Thus, APL1A and LRIM1 can form a disulfide-bonded heterodimer when expressed within dipteran cells. The observed molecular mass of the heterodimer is higher than expected (Fig 6E), which was also observed for the APL1C/LRIM1 complex, presumably due to structural effects of the disulfide bonds [39].

Thus, each of the three APL1 family proteins (APL1A, APL1B and APL1C) can form a heterodimer with LRIM1. LRIM1 protects against both human and rodent malaria, while APL1A and APL1C are required for protection against only human or rodent parasites, respectively. These results suggest that the APL1 paralogs may be pathogen-selective subunits that bind to an obligate LRIM1 partner. The resulting LRR multimer, with fixed (LRIM1) and one of the variable subunits (APL1), could then direct specific pathogen targeting by the immune complex, which also includes a TEP-family effector subunit.

### APL1B is an immune modifier influencing *Plasmodium* oocyst intensity

**Fig 7:**
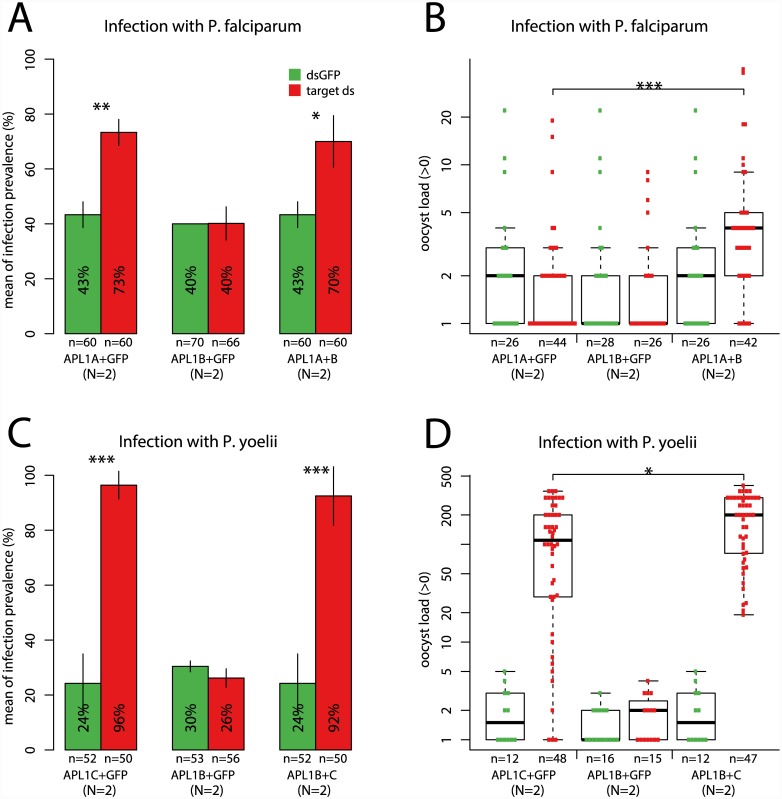
*APL1B* modifies *APL1A* or *APL1C* function, modulating *Plasmodium* infection intensity but not infection prevalence. **A.** *APL1B* activity does not influence permissiveness of mosquitoes to *P*. *falciparum* infection, because no difference in infection prevalence is seen when *APL1B* is silenced alone (*APL1B+GFP*). When *APL1B* is co-silenced with *APL1A*, the infection prevalence is no different than after silencing of *APL1A* alone (*APL1A+B*; detailed statistical comparisons in S3 Table). **B.** *APL1B* influences infection intensity of *P*. *falciparum*, a phenotype detectable only in an *APL1A*-depleted background (*APL1A+B*). **C.** *APL1B* does not influence permissiveness for *P*. *yoelii* infection, because silencing of *APL1B* alone or in combination with *APL1C* has no effect on infection prevalence. **D.** *APL1B* influences infection intensity of *P*. *yoelii*, but the increased infection intensity caused by *APL1B* loss-of-function is only detectable in an *APL1C*-depleted background (*APL1C+B* as compared to *APL1C+GFP*). Graph labels and statistical tests as in Fig 2 legend.

Gene silencing of APL1B alone has not previously revealed a consistent phenotype for this LRR paralog [17, 21, 43]. The results above indicate that APL1B binds LRIM1 (Fig 6B) and belongs to the differentiated Cluster 2 (Fig 1). We wondered whether activity of the other APL1 paralogs might mask the effect of *APL1B* silencing. To re-examine APL1B function, we measured the effect of *APL1B* silencing in a loss-of-function background for either APL1A or APL1C. In an APL1A-depleted background, mosquitoes silenced for *APL1B* displayed elevated *P*. *falciparum* oocyst intensity but not prevalence, as compared to mosquitoes silenced for only *APL1A* alone (Fig 7A and 7B). Similarly for rodent malaria, silencing of *APL1B* in an APL1C-depleted background resulted in higher *P*. *yoelii* oocyst intensity but not prevalence (Fig 7C and 7D, S3 Table). Silencing of *APL1B* alone had no effect upon either parasite (Fig 7). Silencing of APL1B with APL1C depletion produced no phenotype for *P*. *falciparum*, nor did silencing of APL1B with APL1A depletion produce a phenotype for *P*. *yoelii* (S3 Table). Thus, *APL1B* protects mosquitoes against both human and rodent malaria parasites by a mechanism that regulates parasite intensity but not prevalence.

## Discussion

### A population genetic screen for functional immune candidates

Two *Anopheles* immune genes with protective function against malaria parasites in functional assays, *APL1* and *TEP1*, display hallmarks of a coordinate selective sweep in *A*. *coluzzii* as compared to *A*. *gambiae* [14, 15, 27]. We exploited this observation to filter population-based sequences, and found that genes with differentiation signatures similar to the models were highly correlated with functional immune activity. The screen identified novel anti-*Plasmodium* factors in *Anopheles*, characterized their specificity of protection, and revealed new genetic and biochemical interactions among mosquito immune factors.

Our results suggest that the pattern of reduced genetic diversity of immune genes in *A*. *coluzzii* as compared to *A*. *gambiae* is more widespread in the genome than merely *TEP1* and *APL1*. Interestingly, we reanalyzed these 26 genes in an independent set of whole-genome sequenced (WGS) wild mosquitoes from Burkina Faso using the same differentiation metrics as in Fig 1. All but one gene (LRR7059) displayed patterns of differentiation similar to our own data (S3 Fig), despite differences in sequencing technology (manual, Fig 1 and WGS, S3 Fig). The chromosome 3R TEP genes, *TEP12* and *TEP15*, which did not display functional anti-*Plasmodium* activity (Fig 4), also did not display genetic differentiation in the WGS dataset (S3 Fig), consistent with phylogenetic and functional results presented above. This pilot observation suggests that WGS population data from appreciable numbers of sympatric *A*. *gambiae* and *A*. *coluzzii* individuals, when they become available, would be productive to analyze by the same criteria on a genome-wide basis.

Here, we used signatures of population differentiation as a novel line of evidence to prioritize genes with protective immune function when functionally tested against *Plasmodium*. Further work would be necessary to determine their protection profiles against other pathogens. Curiously, the differentiation of these genes in *A*. *coluzzii* is not associated with differential susceptibility to *P*. *falciparum* in nature, because both natural and experimental infection rates of *A*. *gambiae* and *A*. *coluzzii* are equivalent [8, 44–47], despite the striking allele frequency differences between species at the *TEP1* and *APL1* loci [15]. This apparent contradiction between the absence of a detectable allele phenotype in the population and its robust detection by gene silencing might be explained if the different allelic variants for these genes still produce successfully interacting proteins, thus still generating a functional immune complex. In contrast, gene silencing of one essential partner of a functional immune complex would have a dominant effect and entirely abolish the complex. Null alleles for a subunit could also have the same effect as silencing, but null alleles are rare for these genes [14, 15]. Thus, there is no reason to think that the selective sweep in *A*. *coluzzii* generated loss-of-function polymorphism for functional immune complexes, while this is precisely what gene silencing does if targeted to an essential subunit. Further mechanistic studies will be necessary to characterize interacting protein subunits, and specific genetic association studies with appropriate sample sizes and replication would be required to determine whether standing genetic variation at these loci is associated with differential response to *P*. *falciparum* infection in nature.

The selective pressure driving the coordinate sweep in *A*. *coluzzii* is not known. Adult mosquitoes of both species rest together inside village houses, apparently sharing adult ecology, but the two species often occupy larval pools with distinct characteristics [2–4]. Thus, the expansion of *A*. *coluzzii* into a previously uncolonized larval niche likely required adaptation to distinct aquatic microbial communities [14, 15]. The genetic consequence is that *A*. *coluzzii* displays highly simplified allelic spectra for these immune factors, while *A*. *gambiae* maintains high levels of diversity at the same loci [14, 15, 27]. The identities of the putative aquatic microbes that impose differential selection on the two species are unknown. However, the fitness advantage of the presumed new *A*. *coluzzii* niche must be large in order to trade off the loss of immune diversity for a number of immune factors, accompanied by potentially diminished flexibility to respond to new pathogen threats. Thus, a plausible hypothesis that integrates the available observations is that selection of the immune alleles swept in *A*. *coluzzii* relative to *A*. *gambiae* was largely driven by exposure to non-*Plasmodium* environmental microbes enriched in the new *A*. *coluzzii* niche.

We propose that the population screen presented here reveals a network of core immune functions that were selected by prevalent environmental pathogens other than *Plasmodium*, shaping fundamental immune mechanisms that can also be addressed against *Plasmodium*. In fact, evidence is lacking that *Plasmodium* imposes much fitness cost upon infected mosquitoes in nature, and the strongest deleterious effects of *Plasmodium* are seen mainly using artificial host-parasite systems or unnatural infection levels [48–57]. This would also tend to direct attention towards non-*Plasmodium* environmental pathogens as the main evolutionary drivers of immunity in *Anopheles* vectors of malaria. Further functional studies of the sort presented here, clear measurements of the fitness costs of wild *P*. *falciparum* infections, as well as ecological studies to discover important candidate pathogens in the two species’ larval pools, will help to place these observations in context. We suggest that once done, this information will generate important new insight into mechanisms of *Anopheles* immune specificity, the composition of functional immune protein complexes, and the interdependent structure of immune signaling pathways.

### Discovery of novel functional immune factors

The LRR protein, LRIM1, protects *Anopheles* against rodent malaria parasites *P*. *berghei* [35] and *P*. *yoelii* [42], and is required to physically interact with APL1C for protective activity. A previous study did not detect an effect of *LRIM1* silencing on the outcome of *P*. *falciparum* infection [58], whereas our study did assign a significant protective phenotype to *LRIM1* against *P*. *falciparum*. The genetic complexity of wild *Plasmodium* infections can influence the efficiency of mosquito infection [59] and of gene silencing assays [60]. Here, we used the genetically simple laboratory model system of gametocyte culture, which controls for parasite genotype as a variable affecting infection outcome. Two other novel *LRR* genes that we assayed because they were highlighted by their population differentiation (Cluster 2), *LRR7059* and *LRR7037*, displayed functional immune phenotypes, *LRR7059* against *P*. *falciparum* and *LRR7037* against *P*. *yoelii*. Finally, pathogen-specific activity in immunity has not been previously reported for TEP factors, which until now have been regarded as pathogen-general immune effectors. Here, we assayed *TEP3* and *TEP4* because of their differentiation profiles. We find novel pathogen-specific protective functions for the two *TEPs*, which display discrimination for rodent malaria or *P*. *falciparum*, respectively.

A consistent phenotype was not previously detected for the *APL1* paralog, *APL1B*. Here, we detect an activity of *APL1B* only in the absence of function of *APL1A* or *APL1C*. The latter two factors control oocyst infection prevalence, but not oocyst intensity, of human and rodent classes of *Plasmodium*, respectively [21]. In contrast, *APL1B* influences oocyst infection intensity but not prevalence, and does not display a parasite class-specific effect. It thus appears that *APL1A* and *APL1C* control an upstream binary switch for establishment of parasite infection, regardless of intensity. In contrast, *APL1B* may act downstream of these two permissive gates as a rheostat for parasite numbers in successful infections, but without the ability to completely eliminate infection. These results reveal a novel synergistic role for *APL1B* in modulating levels of both human and rodent malaria parasites.

### Immunity and pathogen class

The pathogen class-specific immune molecules (TEP3, APL1A, APL1C, TEP4, LRR7037, LRR7059) may be subunits of combinatorial immune complexes, perhaps conferring functional specificity to the complex. The mechanisms of pathogen-class specificity are unknown. To date, there is no evidence that the mosquito senses *Plasmodium* by recognition of pathogen-associated molecular patterns (PAMPs, [61]). Alternatively, pathogen class-specific responses could be triggered by pathogen behaviors that generate damage-associated molecular patterns (DAMPs, [62]). The presence of enteric microbiota also influences the overall efficiency of *Plasmodium* development in the vector [63–66], and, separately, ookinetes may be labeled for destruction by protein nitration as they traverse epithelial cells [67]. However, it is not evident how these phenomena could be so different for rodent or human malaria in a way that would trigger specific immune signaling and effector responses.

In contrast, the broad-spectrum, class-nonspecific protective molecules (APL1B and LRIM1) may be common or obligatory subunits shared across immune complexes, analogous to the immunoglobulin heavy chain constant domain. APL1B is structurally unique among mosquito immune LRR proteins in its lack of an N-terminal LRR capping motif [68, 69]. This absence is the key attribute that allows APL1B to form homodimers that bind stably to LRIM1, potentially in a higher-order assembly like a 2:2 heterotetramer [23]. The structural biology is consistent with a hypothetical function of the LRIM1/APL1B heterodimer as a common molecular scaffold that can be decorated with functional components that confer specificity for different pathogen classes.

### Conclusion

It is likely that a combinatorial network of functional partners, each with their underlying genetic polymorphism, interacts in order to provide an impressive diversity of immune protection directed against different classes of pathogens. Further work remains to identify the structures and component subunits of immune complexes, the mechanism of pathogen recognition, and the basis of discrimination of pathogen classes. However, we demonstrate that, at least in this system, the use of genetic differentiation resulting from evolutionary divergence between these two closely related species of *Anopheles* is likely to facilitate discovery of yet additional immune genes subject to the same presumptive selection as we exploited in the current study. Further work should also shed light on the nature of the selection pressure driving the differentiation of a number of immune genes in *A*. *coluzzii*.

## Materials and Methods

### Population samples and sequence analysis

Mosquitoes were collected in Goundry, Burkina Faso (12°30'N, 1°20'W), in the Sudan-Savanna ecological zone about 30 km north of Ouagadougou, as described [27, 70]. The samples represent 16 distinct collections from 2007 and 2008. Genomic DNA was isolated, mosquito species determined by standard molecular diagnostics [71] and 24 genes (S1 Table) were PCR amplified and Sanger sequenced from *A*. *gambiae* and *A*. *coluzzii*. Nucleotide diversity (π) and pairwise Fst [27] were computed for *A*. *coluzzii* and *A*. *gambiae* populations (S1 Table) using DnaSP v5 [72]. Nucleotide diversity and Fst data for two additional genes, TEP3 and TEP4, were obtained from published data [15]. The ratio of π in *A*. *coluzzii* to π in *A*. *gambiae* was computed and log transformed. Pairwise Fst was plotted as a function of the log ratio of π. Pi values for all genes in both species are given in S1 Table.

Hierarchical clustering of the gene differentiation data, presented in Fig 1, was done using the Ward method, which minimizes the total within-cluster variance [31]. The Krzanowski-Lai index [32, 33], based on the Within Cluster Sum of Squares (WCSS) as implemented in the NbClust R package, was used to evaluate the goodness of the clustering structure, and reported the optimal number of clusters as k = 3 (S1 Fig).

### Gene silencing assays

The N’gousso mosquito colony, initiated in Cameroon in 2006 [73], was used for functional tests. The colony was formerly described as *A*. *gambiae* fixed for the M molecular form, thus making it *A*. *coluzzii* in the current nomenclature. Double-stranded RNA (dsRNA) was synthesized from the target gene using the T7 Megascript Kit (Ambion) as described previously [21] using indicated primers (S4 Table). For each targeted gene, 200ng of dsRNA (but not more than 207nl, depending on the concentration) were injected into the thorax of cold-anesthetized 1 d-old female mosquitoes using a nano-injector (Nanoject II; Drummond). The efficiency of gene silencing was monitored 4 d after dsRNA injection as follows. After total RNA extraction, cDNA synthesis was performed using M-MLV reverse transcriptase and random hexamers (Invitrogen). For each sample, 1μg of total RNA was used in each of three independent cDNA synthesis reactions. Triplicates were pooled and used as template for qPCR analysis. Real-time PCR was performed using an ABI Prism 7900HT sequence detector (Applied Biosystems). Reactions were prepared in 20 μl volumes using SYBR Green PCR master mix (Applied Biosystems) and 900nM primers with 3 dilutions of cDNA (50, 5 and 0.5 per reaction) in triplicate. S4 Table lists sequences of the primers used for verification of gene silencing. PCR conditions were 95°C for 10 min followed by 40 cycles of 95°C for 15 s, 55°C for 15 s and 60°C for 45 s. mRNA level was normalized to the rpS7 mRNA in each sample and each gene silencing condition was compared to the control using GFP dsRNA.

Calculations were performed using R [74] and the qpcR package [75]. Briefly, raw fluorescence data were normalized in [0,^1^] before fitting a nonlinear sigmoidal model (five-parameter log-logistic model). Efficiency and Ct (defined as the maximum of the second derivative curve) were deduced from the model. The ratio of the normalized gene of interest versus the GFP control was computed using triplicates from the same cDNA dilution, and errors were computed by permutation. For all target genes, mRNA median levels decreased by at least 65% compared to dsGFP controls (S4 Fig).

### *Plasmodium falciparum* gametocyte culture and mosquito infection

*P*. *falciparum* isolate NF54 was cultured using an automated tipper-table system [76] as implemented in the CEPIA mosquito infection core facility of the Institut Pasteur, as previously described [21]. Briefly, a subculture of thawed NF54 stabilate was grown in 10 ml RPMI 1640 medium (PAA), supplemented with 25mM HEPES and L-glutamine, 10% heat-inactivated human serum, and sodium bicarbonate at 0.2% concentration under a constant gas regime (5% CO_2_, 1% O_2_, 94% N_2_). Fresh erythrocytes obtained from blood banks as anonymous samples were added to 7% final concentration. Fourteen days after initiating the subculture, gametocyte maturity was tested by exflagellation of microgametes, and parasitemia and numbers of mature male and female gametocytes were counted on Giemsa stained slides.

For experimental infection of mosquitos, 10 ml of medium containing mature gametocytes was centrifuged at 2000 rpm, and the cell pellet was resuspended in an equal volume of normal type AB human serum. The infected erythrocytes were added to fresh erythrocytes in AB human serum, mixed gently, and transferred to a membrane feeder warmed to 37°C. Mosquitoes were allowed to feed for 15 min, unfed females were discarded and only fully engorged females were used for further analysis. Blood-fed mosquitoes were maintained at 26°C and at 70% relative humidity on 10% sucrose solution supplemented with 0.05% para-amino benzoic acid.

### Rodent malaria infection

Mosquitoes were fed on mice infected with *P*. *berghei* strain PbGFPCON [77], which constitutively expresses green fluorescent protein (GFP), or with *P*. *yoelii yoelii* strain 17XNL, at 8–12% parasitemia with mature gametocytes. Mosquitoes were maintained at 21°C (*P*. *berghei*) or 24°C (*P*. *yoelii*) and 70% relative humidity on 10% sucrose supplemented with 0.05% para-amino benzoic acid. *P*. *yoelii* develops at 24°C, close to the 26°C of *P*. *falciparum*, which minimizes possible temperature effects as compared to the 21°C growth conditions for *P*. *berghei*.

### Analysis of phenotypes

Phenotypes were calculated from biological replicates of ≥20 dissected mosquitoes each. At least two independent replicates were performed for each tested gene for each of at least two different species of *Plasmodium*. Mosquito midguts were dissected at 8 d post-infection. For *P*. *falciparum* and *P*. *yoelii*, midguts were stained with 0.4% mercury dibromofluorescein (Sigma) and the number of oocysts was counted by light microscopy. For *P*. *berghei*, oocysts were counted by fluorescence microscopy. Phenotypes measured were oocyst infection prevalence, which is the proportion of mosquitoes carrying ≥1 oocyst among the total number of dissected mosquitos, and oocyst intensity, which is the oocyst count in mosquitoes with ≥1 oocyst. *P*. *yoelii* infections in mosquitoes silenced for APL1C display high oocyst intensity and low power to detect increase above a median of 200 oocysts [21], which were consequently not used for analysis (relevant to Fig 7C and 7D). Early oocysts of *P*. *yoelii* can also be melanized in the midgut, which provides the additional measurement of *P*. *yoelii* melanization prevalence (the proportion of mosquitoes with ≥1 oocyst melanized oocyst). Melanization of *P*. *falciparum* or *P*. *berghei* is rare [21, 34] and thus not a biologically informative phenotype. For all parasites, infection prevalence of ≥20% was used as the quality control threshold for successful experiments used in analyses. For P. yoelii, the sum of normal and melanized oocyst prevalence is measured.

Differences in infection prevalence were statistically tested using the Chi-Square test, and analysis of oocyst intensity used the Wilcoxon signed rank non-parametric test. Statistical differences in prevalence and intensity were first tested independently for each independent replication replicate as described above and p-values were empirically determined using 10^5^ Monte-Carlo permutations. Following independent statistical tests, the p-values from independent tests of significance were combined using the meta-analytical approach of Fisher [78] when the direction of change of each independent replicate was concordant (e.g., each independent replicate displayed higher infection prevalence than their paired GFP controls). If independent replicates were not concordant, individual replicate p-values are reported. Statistical analyses were done using R [74].

### Interaction of LRIM1 and APL1 in *Trichoplusia* cells

Full-length LRIM1, APL1A, APL1B and APL1C were cloned into the pFastBacI vector (Invitrogen) with C-terminal 6xHis tags. The LRR domains of LRIM1 (residues 1–332), APL1A1 (residues 1–439), APL1B (residues 1–370) and APL1C (residues 1–424 with deletion of residues 26–130 [19]) were also cloned into pFastBacI with C-terminal 6xHis tags. For LRIM1-FLAG, a FLAG tag was inserted directly following the LRR domain (after LRRCT) by replacing the residues DRLIALKRK with DYKDDDDK.

All proteins were expressed using the Bac-to-Bac system (Invitrogen). Spodoptera frugiperda cells (Sf9, Invitrogen) in Sf900-III medium (Invitrogen) were used for the propagation of the baculoviruses while Trichoplusia ni cells (Expression Systems LLC) in ESF921 medium (Expression Systems LLC) were used for large-scale protein expression at 27°C. Infection with baculovirus constructs was performed at a multiplicity of infection (MOI) of 1.0 while harvesting of cells was optimized for each protein and ranged from 40–72 hours post infection (hpi). Anti-FLAG co-immunoprecipitation of APL1 genes with LRIM1 was performed using conditioned media collected 24–72 hpi as previously described [19, 79], and evaluated with reducing or non-reducing 4–20% SDS-PAGE and α6xHis/HRP (Clontech) or αFLAG-M2 (Sigma-Aldrich) western blotting.

### Interaction of LRIM1 and APL1 in *Drosophila* S2 cells

*Drosop*hila S2 cells were cultured in monolayer at 27°C in Schneider’s medium (Invitrogen) supplemented with 10% fetal bovine serum (GIBCO BRL). A plasmid expressing C-terminus strep-tagged APL1A1 was created by modifying pAc5.1V5/Strep:APL1A1 [80] using the following primers:

Forward, 5’ TGGAGCCACCCGCAGTTCGAAAAGTGAGTTTAAACCCGCTGATCA.

Reverse, 5’ GAATTCGTTAGGTCTGTGATTGGCGAG.

The DNA sequence coding for the Strep-tag II is underlined in the forward primer. The amplicon was purified from a 1% agarose gel, self-ligated and grown in *E*. *coli*. A plasmid expressing LRIM1 was created by PCR amplification from *A*. *coluzzii* N’gousso genomic DNA by PCR using the following primers flanking the coding region of LRIM1 gene:

LRIM1-5’EcoRI, TTGAATTCAACGCGAAACGAAAGATGATGTCG.

LRIM1-3’V5XbaI, CCTCTAGATCCCAGCTGGCTCGCTAAATTCTGC.

The amplicon was cloned into the dual His and V5-tag insect expression vector, pAc5.1 V5/His (Invitrogen). S2 cells were co-transfected with the expression vector and the selection vector pCoBlast at a ratio of 20:1 with Effectene transfect reagent (Qiagen). For co-expression, S2 cells were co-transfected with APL1A and LRIM1 expression vectors and pCoBlast in a ratio 10:10:1. Three days after transfection, antibiotic selection was started with 25 μg/ml blasticidin (Invitrogen). Medium with blasticidin was changed every 3 d until antibiotic resistant cells appeared (typically in 1.5 weeks). After 3 weeks, the cells were tested for protein expression. Strep-tagged proteins in the culture supernatant were purified by 500 μl bed volume of Strep-Tactin superflow plus (Qiagen) with a gravity flow column according to the manufacturer’s instruction (Qiagen). Briefly, following centrifugation (3000 x g for 10 min at 4°C) the culture supernatant was applied to the column, and then washed with 10 ml of buffer NP (50 mM NaH_2_PO_4_, 300 mM NaCl, pH 8.0). The bound proteins were eluted with 1 ml of buffer NPD (50 mM NaH_2_PO_4_, 300 mM NaCl, 2.5 mM desthiobiotin, pH 8.0).

Proteins were separated by SDS-PAGE (12.5% Criterion Tris-HCl gel, Bio-Rad) and stained with Bio-safe Coomassie Stain (Bio-Rad). For Western blotting, the SDS-PAGE gel was transferred to Amersham Hybond-P PVDF membrane (GE). Blots were incubated with Tris buffered saline with Tween 20 (TBST) containing 2.5% (w/v) skim milk and 0.05% (v/v) mouse anti-strep-tag classic (AbD Serotec) or 0.05% (v/v) Anti-V5 mouse monoclonal antibody (Invitrogen) for 1 h at RT, following blocking the membrane with TBST 2.5% skim milk for 1h at RT. Blots were washed 3 times with TBST, incubated with TBST 2.5% skim milk containing goat anti-mouse IgG1 horseradish peroxidase conjugate (Invitrogen) for 1h at RT, washed 3 times with TBST, and then visualized by SuperSignal West Pico Chemiluminescent Substrate (Thermo scientific).

### Ethics statement

There were no human subjects. The protocol for the ethical treatment of the animals used in this study was approved by the research animal ethics committee of the Institut Pasteur, “C2EA-89 CETEA Institut Pasteur” as protocol number B75-15-31. The Institut Pasteur ethics committee is authorized by the French Ministry of Higher Education and Research (MESR) under French law N° 2001–486, which is aligned with Directive 2010/63/EU of the European Commission on the protection of animals used for scientific purposes.

## Supporting Information

S1 Table Gene list and cluster composition. (DOCX)

S2 Table Statistical analysis of infection phenotypes following gene silencing. Summary data for all experimental replicates testing the effect of target gene silencing compared to control treatment with dsGFP. Statistically significant differences are indicated by green shading. Row indicates target gene tested (injected dsRNA) and challenge parasite (infection) for a given replicate, with the corresponding replicates indicated in the following row(s) for the same target gene and parasite. Individual p-values were calculated per replicate by statistical comparison to the control experiment indicated in the same row (control experiment #), which refers to the corresponding dsGFP control experiment shown in column 1 (experiment #). If the replicates of a gene/parasite test were consistent (in the same phenotypic direction, see Methods), then the individual p-values were combined by Fisher's method (Fisher combined prob). If the replicate phenotypes were not consistent, the individual p-values are shown but combining of p-values is not justified. NA, Not Appropriate, combining of individual p-values not justified; NM, Not Measured, specific phenotype not measured because infrequent. Data presented in this table is summarized in graphical form in Fig 5.
(XLSX)

S3 Table Statistical analysis of APL1B effect in the context of APL1C or APL1A loss of function. Mosquitoes treated with dsAPL1B+dsAPL1A and dsAPL1B+dsAPL1C are compared to mosquitoes treated with only dsAPL1A or dsAPL1C, respectively. Statistically significant differences are indicated by green shading. Row indicates target gene tested (injected dsRNA) and challenge parasite (infection) for a given replicate, with the corresponding replicates indicated in the following row(s) for the same target gene and parasite. Individual p-values were calculated per replicate by statistical comparison to the control experiment indicated in the same row (control experiment #), which refers to the corresponding dsAPL1A or ds APL1C control experiment shown in column 1 (experiment #). If the replicates of a gene/parasite test were consistent (in the same phenotypic direction, see Methods), then the individual p-values were combined by Fisher's method (Fisher combined prob). If the replicate phenotypes were not consistent, the individual p-values are shown but combining of p-values is not justified. Experiment numbers (column 1) correspond to S2 Table, where the same replicate experiments were analyzed by comparison to dsGFP-treated mosquitoes as controls. NA, Not Appropriate, combining of individual p-values not justified; NM, Not Measured, specific phenotype not measured because infrequent.
(XLSX)

S4 Table Primer sequences. Sequences of the primers used for synthesis of double-stranded RNA (ds synthesis) and for gene silencing validation (gs validation) of target genes by qPCR. T7 sequence is underlined.
(DOCX)

S1 Fig Optimal clustering of gene sequence data points shown in Fig 1. The Krzanowski-Lai index (see Material and Methods) based on the Within Cluster Sum of Squares (WCSS) as implemented in the NbClust R package was used to assess the goodness of the clustering structure without respect to external information. The optimal number of clusters (maximizing the Krzanowski-Lai index) was k = 3.
(DOCX)

S2 Fig Phylogeny of *Anopheles gambiae* TEP proteins. Two genomic TEP gene groups are located on chromosomes 3R and 3L, respectively. Predicted protein sequences were aligned and a neighbor joining tree computed using Clustal X. Bootstrap values were obtained with 1000 trials. TEP proteins that were functionally tested in this study are highlighted in red (TEP3, TEP4) and green (TEP12, TEP15).
(DOCX)

S3 Fig Whole-genome sequence identifies similar patterns of population genetic differentiation. Data corresponding to the 28 studied genes were extracted from a set of 81 *A*. *coluzzii* and 84 *A*. *gambiae* wild mosquitoes from Burkina Faso that were whole-genome sequenced by the *Anopheles gambiae* 1000 Genomes (Ag1k) project at the Wellcome Trust Sanger Institute (VCF files from pre-publication data release provided by kind permission of the Ag1k project. Source: The *Anopheles gambiae* 1000 Genomes Consortium (2014): Ag1000G phase 1 AR2 data release. MalariaGEN. http://www.malariagen.net/data/ag1000g-phase1-AR2). The same methods of analysis were applied as those described in Methods for Fig 1. This result replicates the main analysis using an independent sample set from a different site sympatric for *A*. *coluzzii* and *A*. *gambiae*, using nucleotide variation data generated by a different sequencing technology. Patterns of gene sequence differentiation are strikingly similar, with all but one gene (LRR7059) exhibiting patterns of differentiation similar to those detected by manual Sanger sequencing of *A*. *coluzzii* and *A*. *gambiae* samples in the current study (Fig 1).
(DOCX)

S4 Fig Verification of gene silencing. Relative quantification of gene expression in RNAi-mediated gene silencing experiments, using expression of the ribosomal protein rps7 gene as the internal calibrator. For all cases, mRNA median levels of the targeted gene decreased by at least 65%. Analysis was performed using R [74] and the qpcR package [75]. A nonlinear sigmoidal model (five-parameter log-logistic model) was fit to normalized fluorescence data and efficiency and Ct (defined as the maximum of the second derivative curve) were deduced from the model. The ratio of the normalized gene of interest versus the GFP control was computed using triplicates from the same cDNA dilution. Error bars show median absolute deviation computed by permutation.
(DOCX)
