# Correction: An Evolution-Based Screen for Genetic Differentiation between Anopheles Sister Taxa Enriches for Detection of Functional Immune Factors

**DOI:** 10.1371/journal.ppat.1005836

**Published:** 2016-08-12

**Authors:** Christian Mitri, Emmanuel Bischoff, Eizo Takashima, Marni Williams, Karin Eiglmeier, Adrien Pain, Wamdaogo M. Guelbeogo, Awa Gneme, Emma Brito-Fravallo, Inge Holm, Catherine Lavazec, N’Fale Sagnon, Richard H. Baxter, Michelle M. Riehle, Kenneth D. Vernick

Fig 6 is incorrect. In panel B, the image of the experimental result from the top panel was duplicated into the frame of the bottom panel in place of the image for the control. The authors have provided a corrected version here.

**Fig 6:**
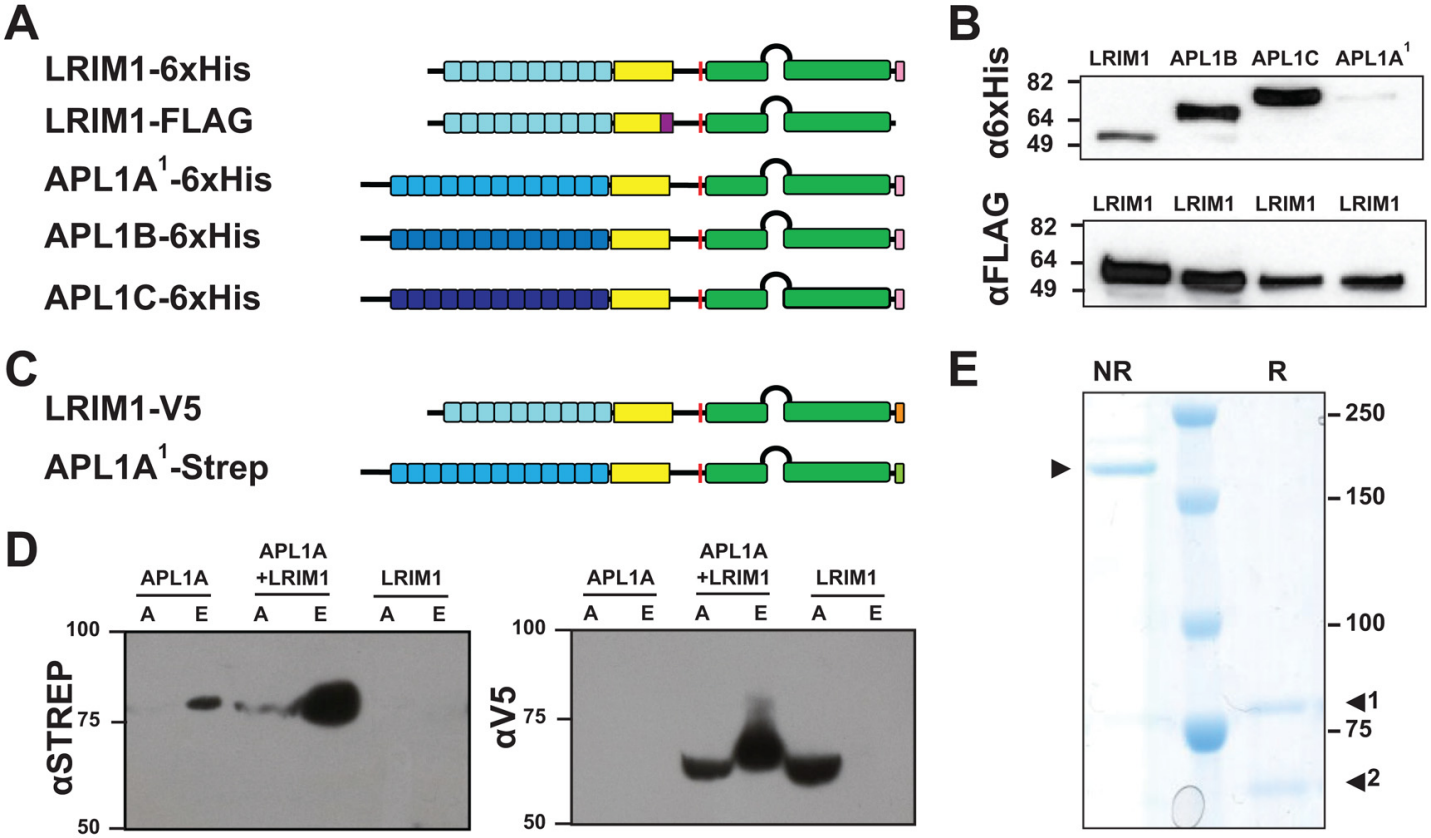
LRIM1 can form protein complexes with all members of the APL1 family. **A.** Structure of expression plasmids used for co-immunoprecipitation of proteins expressed in *Trichoplusia* cells. Fusion tags: purple, FLAG tag; pink, 6xHis tag. Small boxes represent LRR repeats; yellow, Cys-rich region; green, coiled-coil (CC) domain with helix-loop-helix region between CC domains. Additional structural details in [23]. **B.** FLAG-tagged LRIM1 was used to pull down 6xHis-tagged LRIM1, APL1A1, APL1B and APL1C. Western blots were performed with anti-6xHis/HRP (top panel) and anti-FLAG/HRP (bottom panel) to detect the 6xHis-tagged co-precipitated proteins and FLAG-tagged LRIM1, respectively. **C.** Expression plasmids used for pull-downs of proteins expressed in *Drosophila* S2 cells. Fusion tags: orange, V5 tag; light green, Strep tag; other protein features as in (A). **D.** S2 cells were transfected with Strep-tagged APL1A, V5-tagged LRIM1, or co-transfected with both plasmids. Cell extracts were purified by Strep-tag column affinity, and analyzed by Western blot using anti-Strep or anti-V5 antibodies. Lane labels: A, cell extract applied to Strep column; E, eluted fraction from Strep column. **E.** Coomassie-stained SDS-PAGE of extract from co-transfected cells (APL1A-Strep+LRIM1-V5) after elution from Strep column. Lane NR, sample non-reduced, arrow indicates APL1A/LRIM1 heterodimer; lane R, sample reduced, arrow 1 indicates APL1A-Strep monomer, arrow 2 LRIM1-V5 monomer.
