# Supplementary material for: An Evolution-Based Screen for Genetic Differentiation between Anopheles Sister Taxa Enriches for Detection of Functional Immune Factors
Source: PLoS Pathog. 2015 Dec 3;11(12):e1005306. doi: 10.1371/journal.ppat.1005306 (PMC4669117; doi:10.1371/journal.ppat.1005306)
Supplement: S1 Table — (DOCX) [file ppat.1005306.s001.docx]

**S1 Table. Gene list and cluster composition.**

| *geneID* | *gene name* | *M^a^* | *S^a^* | *Fst* | *π_M_ ^b^* | *π_S_^b^* | *log_10_ (π_M_/π_S_)* | *cluster* | *dataset ^d^* |
| --- | --- | --- | --- | --- | --- | --- | --- | --- | --- |
| AGAP005681 | GPRNNA21 | 32 | 60 | 0.085 | 0.007 | 0.007 | 0.015 | 3 | Crawford |
| AGAP005693 | APL2 | 32 | 60 | 0.011 | 0.009 | 0.009 | 0.005 | 3 | Crawford |
| AGAP005716 | SCRB16 | 32 | 60 | 0.047 | 0.019 | 0.018 | 0.008 | 3 | Crawford |
| AGAP005728 | 5728 | 32 | 60 | 0.009 | 0.005 | 0.006 | -0.044 | 3 | Crawford |
| AGAP005762 | 5762 | 32 | 59 | 0.050 | 0.022 | 0.030 | -0.130 | 3 | Crawford |
| AGAP006102 | PRS1 | 32 | 60 | 0.002 | 0.012 | 0.014 | -0.062 | 3 | Crawford |
| AGAP006348 | LRIM1 | 32 | 60 | 0.308 | 0.014 | 0.019 | -0.152 | 2 | Crawford |
| AGAP006421 | IRSP1 | 32 | 60 | 0.044 | 0.015 | 0.022 | -0.167 | 3 | Crawford |
| AGAP006974 | TOLL9 | 32 | 60 | 0.005 | 0.033 | 0.030 | 0.042 | 3 | Crawford |
| AGAP007030 | LRR7030 | 32 | 59 | 0.105 | 0.018 | 0.025 | -0.144 | 3 | Crawford |
| AGAP007032 | 7032 | 32 | 60 | 0.015 | 0.022 | 0.020 | 0.044 | 3 | Crawford |
| AGAP007033 | APL1C | 32 | 58 | 0.435 | 0.014 | 0.031 | -0.356 | 2 | Crawford |
| AGAP007034 | LRR7034 | 31 | 60 | 0.133 | 0.007 | 0.006 | 0.094 | 3 | Crawford |
| AGAP007036 | APL1A | 31 | 55 | 0.517 | 0.033 | 0.070 | -0.325 | 2 | Crawford |
| AGAP007037 | LRR7037 | 32 | 60 | 0.104 | 0.003 | 0.007 | -0.437 | 2 | Crawford |
| AGAP007041 | FBN32 | 94 | 101 | 0.050 | 0.012 | 0.014 | -0.073 | 3 | Crawford |
| AGAP007048 | LRR7048 | 94 | 100 | 0.037 | 0.013 | 0.013 | 0.003 | 3 | Crawford |
| AGAP007058 | DLL | 94 | 101 | 0.019 | 0.013 | 0.016 | -0.106 | 3 | Crawford |
| H603 flanking | intergenic region | 94 | 100 | 0.005 | 0.012 | 0.015 | -0.119 | 3 | Crawford |
| AGAP007059 | LRR7059 | 94 | 101 | 0.168 | 0.007 | 0.014 | -0.296 | 2 | Crawford |
| AGAP007060 | LRR7060 | 94 | 101 | 0.058 | 0.010 | 0.010 | -0.002 | 3 | Crawford |
| AGAP007061 | LRR7061 | 32 | 60 | 0.046 | 0.014 | 0.011 | 0.094 | 3 | Crawford |
| AGAP010815 | TEP1 | 32 | 60 | 0.545 | 0.001 | 0.006 | -0.885 | 1 | Crawford |
| AGAP007035 | APL1B | 19 | 12 | 0.419 | 0.014 | 0.039 | -0.442 | 2 | Rottschaefer |
| AGAP010816 | TEP3 | 22 | 16 | 0.617 | 0.001 | 0.004 | -0.579 | 1 | White |
| AGAP010812 | TEP4 | 22 | 16 | 0.221 | 0.006 | 0.012 | -0.288 | 2 | White |

^a^ number of individual sequences analyzed from M (*A. coluzzii*) and S (*A. gambiae*) mosquitoes

^b^ Nucleotide diversity in *A. coluzzii* population.

^c^ Nucleotide diversity in *A. gambiae* population.

^d^ references:

Crawford, J.E., Bischoff, E., Garnier, T., Gneme, A., Eiglmeier, K., Holm, I., Riehle, M.M., Guelbeogo, W.M., Sagnon, N., Lazzaro, B.P., and Vernick, K.D. (2012). Evidence for Population-Specific Positive Selection on Immune Genes of *Anopheles* *gambiae*. G3 Genes Genomes Genetics 2, 1505-1519.

Rottschaefer S. M., Riehle M. M., Coulibaly B., Sacko M., Niaré O. et al., 2011 Exceptional diversity, maintenance of polymorphism, and recent directional selection on the APL1 malaria resistance genes of *Anopheles* *gambiae*. PLoS Biol. 9: e1000600.

White B. J., Lawniczak M. K., Cheng C., Coulibaly M. B., Wilson M. D. et al., 2011 Adaptive divergence between incipient species of *Anopheles* *gambiae* increases resistance to Plasmodium. Proc. Natl. Acad. Sci. U. S. A. 108: 244-249.
