# Supplementary material for: An Evolution-Based Screen for Genetic Differentiation between Anopheles Sister Taxa Enriches for Detection of Functional Immune Factors
Source: PLoS Pathog. 2015 Dec 3;11(12):e1005306. doi: 10.1371/journal.ppat.1005306 (PMC4669117; doi:10.1371/journal.ppat.1005306)
Supplement: S4 Table — Sequences of the primers used for synthesis of double-stranded RNA (ds synthesis) and for gene silencing validation (gs validation) of target genes by qPCR. T7 sequence is underlined. (DOCX) [file ppat.1005306.s004.docx]

**S4 Table. Primer sequences.** Sequences of the primers used for synthesis of double-stranded RNA (ds synthesis) and for gene silencing validation (gs validation) of target genes by qPCR. T7 sequence is underlined.

| **Primer name** | **Function** | **Sequence** |
| --- | --- | --- |
| T7-GFP-F | ds synthesis | TAATACGACTCACTATAGGGCATGGTGAGCAAGGGCGAG |
| T7-GFP-R | ds synthesis | TAATACGACTCACTATAGGGCTTACTTGTACAGCTCGTC |
| T7-APL1A-F | ds synthesis | TAATACGACTCACTATAGGACTACCACCAGCCGAAAGATG |
| T7-APL1A-R | ds synthesis | TAATACGACTCACTATAGGATCTGGTCTTGTATAGTACAATGG |
| T7-APL1B-F | ds synthesis | TAATACGACTCACTATAGGACTCGCAAAGCTCAGCAAACAC |
| T7-APL1B-R | ds synthesis | TAATACGACTCACTATAGGAGTGAGAACAAATAAGTTCAAAGTCC |
| T7-APL1C-F | ds synthesis | TAATACGACTCACTATAGGAGGCCAAGAAGAACCGCAATCC |
| T7-APL1C-R | ds synthesis | TAATACGACTCACTATAGGATCACAGTGATTTCAGGGTGTGC |
| T7-APL2-F | ds synthesis | TAATACGACTCACTATAGGGCGGGATGTCATTCACTCACG |
| T7-APL2-R | ds synthesis | TAATACGACTCACTATAGGGCGGCATTTCAAAAATTACCC |
| T7-LRIM1-F | ds synthesis | TAATACGACTCACTATAGGGCTGGAACGTAAAGGAGCTTG |
| T7-LRIM1-R | ds synthesis | TAATACGACTCACTATAGGGCGCTCGGCAAAGTTCACCGT |
| T7-LRR7037-F | ds synthesis | TAATACGACTCACTATAGGGCCGTTGTCCAGTATCCACAG |
| T7-LRR7037-R | ds synthesis | TAATACGACTCACTATAGGGCCAACAACACGATCAAGCAG |
| T7-LRR7048-F | ds synthesis | TAATACGACTCACTATAGGGCTTTTTAAGCCTAGCCCGTCTG |
| T7-LRR7048-R | ds synthesis | TAATACGACTCACTATAGGGCGCAGCTCGGTAAGCCGATTG |
| T7-7058-F | ds synthesis | TAATACGACTCACTATAGGGCGTTAAGATCTGGTTTCAAAATCG |
| T7-7058-R | ds synthesis | TAATACGACTCACTATAGGGCGTACGTAGCCACCCATCTGC |
| T7-LRR7059-R | ds synthesis | TAATACGACTCACTATAGGGCACCAGGCGCTAGTTCTTTGA |
| T7-LRR7059 -F | ds synthesis | TAATACGACTCACTATAGGGCTACCGGCAACGGTCTTTAAC |
| T7-LRR7060-F | ds synthesis | TAATACGACTCACTATAGGGCGAAGCACTTCCACTGGTGCT |
| T7-LRR7060-R | ds synthesis | TAATACGACTCACTATAGGGCAGTAGCAGGCTCGTGAGTGAG |
| T7-LRR7061-F | ds synthesis | TAATACGACTCACTATAGGGCACGCAAGGTGGAAAAGCTGT |
| T7-LRR7061-R | ds synthesis | TAATACGACTCACTATAGGGCTCCTTCAGCCGCTGATTGTT |
| rpS7-F | gs validation | ATGGTGGTCTGCTGGTTCTT |
| rpS7-R | gs validation | CACCGCCGTGTACGATGCCA |
| APL1A-VF | gs validation | GACTGCAAGCCGAGATCGATACC |
| APL1A-VR | gs validation | CATCCATCTGGTCCTTGAGCTTA |
| APL1B-VF | gs validation | CTGGAACAGGAAAACATTGCAC |
| APL1B-VR | gs validation | TACAGTCGAACCGCCCTAGATG |
| APL1C-VF | gs validation | AAGCAGGCTGAGTTGAGACAGG |
| APL1C-VR | gs validation | GCCCAAGTAACATCATACACAC |
| APL2-VF | gs validation | ATGGTTAAACTGTGGGCAATCG |
| APL2-VR | gs validation | CTACGGTCCAGCTCCATTGTG |
| LRIM1-VF | gs validation | GTGCCAAGTCGTCCTATTGCTC |
| LRIM1-VR | gs validation | TGCTGTCCGTTACCTTCTCGAT |
| LRR7037-VF | gs validation | TGCAGGAGCTGCACCTGTACG |
| LRR7037-VR | gs validation | GCTGCTTGATCGTGTTGTTG |
| LRR7048-VF | gs validation | CGAACCGGAAGCTGGTGGAGC |
| LRR7048-VR | gs validation | GGCAGCTCGGTAAGCCGATTG |
| 7058-VF | gs validation | CCAGCTGCAGCAGCTCAACCG |
| 7058-VR | gs validation | CGATTTTGAAACCAGATCTTA |
| LRR7059-VF | gs validation | TAGATACCTTTTCGGCAGAGG |
| LRR7059-VR | gs validation | AGGCGCTAGTTCTTTGATCTG |
| LRR7060-VF | gs validation | AGTACACGTTCGAGGGGTTGG |
| LRR7060-VR | gs validation | GTAGCAGGCTCGTGAGTGAGC |
| LRR7061-VF | gs validation | GCTTCGCGGGGCTGTGGAAGC |
| LRR7061-VR | gs validation | GCGACAGCTTTTCCACCTTGC |
| T7-Tep3-F | ds synthesis | TAATACGACTCACTATAGGGCACCTCGACTGAGAAAGGTTTG |
| T7-Tep3-R | ds synthesis | TAATACGACTCACTATAGGGCTGATTATTTATATAGTTTTAC |
| T7-Tep4-F | ds synthesis | TAATACGACTCACTATAGGGCGGCGGAAAAGATCTCCCCG |
| T7-Tep4-R | ds synthesis | TAATACGACTCACTATAGGGCGCGGCCGTCCGACAGCTGCG |
| T7-Tep12-F | ds synthesis | TAATACGACTCACTATAGGGCGAGTGATCCCAACTCAAAAC |
| T7-Tep12-R | ds synthesis | TAATACGACTCACTATAGGGCTTACCCGAGGAAAATAAA |
| T7-Tep15-F | ds synthesis | TAATACGACTCACTATAGGGCTGAACGCGAACACCAATCC |
| T7-Tep15-R | ds synthesis | TAATACGACTCACTATAGGGCCGGATGCTGCACGCCTCACC |
| Tep3-VF | gs validation | ACCGCCAGGCGTACGTGATGG |
| Tep3-VR | gs validation | CAAACCTTTCTCAGTCGAGGT |
| Tep4-VF | gs validation | GGACCTCCATAATGCGGTGGC |
| Tep4-VR | gs validation | CGGGGAGATCTTTTCCGCCAG |
| Tep12-VF | gs validation | TAAGTGTCTCGTTGATTTCGC |
| Tep12-VR | gs validation | GTTTTGAGTTGGGATCACTC |
| Tep15-VF | gs validation | GGCCACGGGCAGTGGTTTTGC |
| Tep15-VR | gs validation | GGATTGGTGTTCGCGTTCACC |
