# Supplementary material for: An Evolution-Based Screen for Genetic Differentiation between Anopheles Sister Taxa Enriches for Detection of Functional Immune Factors
Source: PLoS Pathog. 2015 Dec 3;11(12):e1005306. doi: 10.1371/journal.ppat.1005306 (PMC4669117; doi:10.1371/journal.ppat.1005306)
Supplement: S1 Fig — The Krzanowski-Lai index (see Material and Methods) based on the Within Cluster Sum of Squares (WCSS) as implemented in the NbClust R package was used to assess the goodness of the clustering structure without respect to external information. The optimal number of clusters (maximizing the Krzanowski-Lai index) was k = 3. (DOCX) [file ppat.1005306.s005.docx]

**S1 Fig. Optimal clustering of gene sequence data points shown in Fig 1.** The Krzanowski-Lai index (see Material and Methods) based on the Within Cluster Sum of Squares (WCSS) as implemented in the NbClust R package was used to assess the goodness of the clustering structure without respect to external information. The optimal number of clusters (maximizing the Krzanowski-Lai index) was k=3.
