# Supplementary material for: An Evolution-Based Screen for Genetic Differentiation between Anopheles Sister Taxa Enriches for Detection of Functional Immune Factors
Source: PLoS Pathog. 2015 Dec 3;11(12):e1005306. doi: 10.1371/journal.ppat.1005306 (PMC4669117; doi:10.1371/journal.ppat.1005306)
Supplement: S2 Fig — Two genomic TEP gene groups are located on chromosomes 3R and 3L, respectively. Predicted protein sequences were aligned and a neighbor joining tree computed using Clustal X. Bootstrap values were obtained with 1000 trials. TEP proteins that were functionally tested in this study are highlighted in red (TEP3, TEP4) and green (TEP12, TEP15). (DOCX) [file ppat.1005306.s006.docx]

**S2 Fig. Phylogeny of *Anopheles* *gambiae* TEP proteins.** Two genomic TEP gene groups are located on chromosomes 3R and 3L, respectively. Predicted protein sequences were aligned and a neighbor joining tree computed using Clustal X. Bootstrap values were obtained with 1000 trials. TEP proteins that were functionally tested in this study are highlighted in red (TEP3, TEP4) and green (TEP12, TEP15).
