# Supplementary material for: An Evolution-Based Screen for Genetic Differentiation between Anopheles Sister Taxa Enriches for Detection of Functional Immune Factors
Source: PLoS Pathog. 2015 Dec 3;11(12):e1005306. doi: 10.1371/journal.ppat.1005306 (PMC4669117; doi:10.1371/journal.ppat.1005306)
Supplement: S3 Fig — Data corresponding to the 28 studied genes were extracted from a set of 81 A. coluzzii and 84 A. gambiae wild mosquitoes from Burkina Faso that were whole-genome sequenced by the Anopheles gambiae 1000 Genomes (Ag1k) project at the Wellcome Trust Sanger Institute (VCF files from pre-publication data release provided by kind permission of the Ag1k project. Source: The Anopheles gambiae 1000 Genomes Consortium (2014): Ag1000G phase 1 AR2 data release. MalariaGEN. http://www.malariagen.net/data/ag1000g-phase1-AR2). The same methods of analysis were applied as those described in Methods for Fig 1. This result replicates the main analysis using an independent sample set from a different site sympatric for A. coluzzii and A. gambiae, using nucleotide variation data generated by a different sequencing technology. Patterns of gene sequence differentiation are strikingly similar, with all but one gene (LRR7059) exhibiting patterns of differentiation similar to those detected by manual Sanger sequencing of A. coluzzii and A. gambiae samples in the current study (Fig 1). (DOCX) [file ppat.1005306.s007.docx]

**S3 Fig. Whole-genome sequence identifies similar patterns of population genetic differentiation.** Data corresponding to the 28 studied genes were extracted from a set of 81 *A. coluzzii* and 84 *A. gambiae* wild mosquitoes from Burkina Faso that were whole-genome sequenced by the *Anopheles* *gambiae* 1000 Genomes (Ag1k) project at the Wellcome Trust Sanger Institute (VCF files from pre-publication data release provided by kind permission of the Ag1k project. Source: The *Anopheles* *gambiae* 1000 Genomes Consortium (2014): Ag1000G phase 1 AR2 data release. MalariaGEN. http://www.malariagen.net/data/ag1000g-phase1-AR2). The same methods of analysis were applied as those described in Methods for Fig 1. This result replicates the main analysis using an independent sample set from a different site sympatric for *A. coluzzii* and *A. gambiae*, using nucleotide variation data generated by a different sequencing technology. Patterns of gene sequence differentiation are strikingly similar, with all but one gene (LRR7059) exhibiting patterns of differentiation similar to those detected by manual Sanger sequencing of *A. coluzzii* and *A. gambiae* samples in the current study (Fig 1).
