# Supplementary material for: An Evolution-Based Screen for Genetic Differentiation between Anopheles Sister Taxa Enriches for Detection of Functional Immune Factors
Source: PLoS Pathog. 2015 Dec 3;11(12):e1005306. doi: 10.1371/journal.ppat.1005306 (PMC4669117; doi:10.1371/journal.ppat.1005306)
Supplement: S4 Fig — Relative quantification of gene expression in RNAi-mediated gene silencing experiments, using expression of the ribosomal protein rps7 gene as the internal calibrator. For all cases, mRNA median levels of the targeted gene decreased by at least 65%. Analysis was performed using R [74] and the qpcR package [75]. A nonlinear sigmoidal model (five-parameter log-logistic model) was fit to normalized fluorescence data and efficiency and Ct (defined as the maximum of the second derivative curve) were deduced from the model. The ratio of the normalized gene of interest versus the GFP control was computed using triplicates from the same cDNA dilution. Error bars show median absolute deviation computed by permutation. (DOCX) [file ppat.1005306.s008.docx]

**S4 Fig. Verification of gene silencing.** Relative quantification of gene expression in RNAi-mediated gene silencing experiments, using expression of the ribosomal protein rps7 gene as the internal calibrator. For all cases, mRNA median levels of the targeted gene decreased by at least 65%. Analysis was performed using R ([R Core Team, 2012](#_ENREF_49)) and the qpcR package ([Ritz and Spiess, 2008](#_ENREF_54)). A nonlinear sigmoidal model (five-parameter log-logistic model) was fit to normalized fluorescence data and efficiency and Ct (defined as the maximum of the second derivative curve) were deduced from the model. The ratio of the normalized gene of interest versus the GFP control was computed using triplicates from the same cDNA dilution. Error bars show median absolute deviation computed by permutation.
